# Learning deficits and early school leaving: Evidence from a longitudinal study in India

**DOI:** 10.1371/journal.pone.0336850

**Published:** 2025-11-18

**Authors:** K. G. Santhya, Nicole A. Haberland, A. J. Francis Zavier

**Affiliations:** 1 Independent researcher, Kollam, India,; 2 Department of Population and Family Health, Columbia University Mailman School of Public Health, New York, New York, United States of America; 3 Population Research Centre, Gandhigram, India; Jawaharlal Nehru University, INDIA

## Abstract

Although the relationship between learning deficits (LD) and early school leaving (ESL) is extensively acknowledged in studies from the Global North, fewer studies from the Global South have examined this relationship. We examined the levels and patterns of ESL among adolescents, relationship between LD and ESL and the gender dimensions, if any, in this relationship in India. We used data from a state-representative longitudinal study of adolescents aged 10−19 in Bihar and Uttar Pradesh states of India, conducted in 2015−16 and 2018−19. Descriptive analysis drew on data from adolescents ever enrolled in school (N = 11,476) and multivariate analyses used data from adolescents enrolled in school at wave 1 (N = 9,169). We used discrete-time hazard and fixed effects regression models to examine the relationship between LD and ESL. The probability of ESL was 39%, with a higher probability for girls (42%) than boys (38%). Although learning levels improved over time, 53% of adolescents displayed moderate or severe LD. Discrete-time hazard models show that LD influenced the probability of ESL (β = 1.959, p < 0.001 for those with severe LD and β = 0.568, p < 0.001 for those with moderate LD). Learning deficits equally affected the probability of ESL among girls and boys. Fixed effects regression models reiterate these findings. Investment in improving foundational skills is paramount for preventing early school leaving for girls and boys. However, the potential benefits will only be fully realised with accompanying measures which address gendered beliefs and practices and premature transition to adult roles, enhance parental engagement and improve education systems.

## Introduction

Early school leaving and extensive learning deficits remain critical challenges in global education, with significant implications for youth development and societal progress. Despite notable advances in expanding access to education, completion rates in 2023 remained far below SDG targets, with 85% of children completing primary school, 78% completing lower secondary school and only 59% completing upper secondary school worldwide [1]. Moreover, 42% of youth aged 21–24 globally were classified as early school leavers, that is, they left schooling without completing 12 years of education [2]. Learning outcomes are equally concerning, with an estimated 70% of children in learning poverty (i.e., being unable to read and understand a simple text by age 10) in low- and middle-income countries in 2022 [3]. The economic costs of early school leaving and learning deficits are massive [2]. Early school leaving and learning deficits also have substantial social and health consequences, including the risk of early marriage and childbearing, poor mental health, homicides and other forms of violence [4,5].

The relationship between learning deficits and early school leaving is extensively acknowledged in the global literature, particularly in studies from the Global North [6–11]. Poor academic performance, although defined and measured differently, for example, low academic achievement, grade repetition, lack of interest in classes, low learning skills, low IQ or learning difficulties among others, is found to be a strong predictor of school discontinuation. However, studies that explored this relationship, particularly the causal relationship, are few and far between in the Global South. This is partially because of limited availability of studies that used panel designs that are a better fit for exploring school discontinuation as a longer process of disengagement [12] and studies that measured learning deficits objectively. Moreover, endogeneity issues arising from simultaneity, omitted variables and measurement errors complicate assessing this relationship [13].

The gendered dimension of factors underlying school participation and performance is documented in a few studies in the Global South. For example, a study that explored the effect of primary school quality on school discontinuation among Kenyan girls and boys found that the quality of the school environment had a larger impact on discontinuation among girls than boys [14]. The study reported that girls attending schools in which boys were favoured, where teachers gave lesser importance of hard subjects for girls and where boys were not restrained from harassing girls had higher odds of discontinuing school than boys. Another study in Ethiopia and Guinea observed that adverse cultural practices operating at the household, school, labour market and societal levels affected the enrolment and performance of girls more than boys [15]. Similarly, a study from India reported that positive classroom dynamics was correlated with academic performance of girls more than boys [16]. However, whether learning deficits have similar or differential impact on early school leaving among girls and boys is less researched in the Global South.

As elsewhere in the Global South, only a few studies examined the relationship between educational performance and school discontinuation in India [13,17–21]. Although measured differently (e.g., grade repetition, perceptions about performance, receptive vocabulary ability test, Raven’s test, reading skill, test in mathematics and Hindi), most of these studies confirm a direct relationship between learning deficits and school discontinuation. Even so, findings are not consistent across studies. While Paul and co-authors’ [21] analysis of data from two rounds of the India Human Development Survey found that grade repetition increased the risk of the school discontinuation during the inter-survey period, no such relationship was observed in Cueto and León’s [20] analysis of data from five rounds of the Young Lives study in Andhra Pradesh and Telangana. Similarly, while various analyses using Young Lives data showed that reading/writing skills at age 8/12 were associated with the reduced chances of discontinuation [13,18,20], Siddhu [17] observed no such association in a study in Uttar Pradesh. Findings from sex-disaggregated analysis of the association between performance and school discontinuation are also inconsistent. For example, Siddhu [17] found that cognitive ability as assessed by the Raven’s test affected girls’ transition to secondary school but not boys’ transition. On the other hand, Nakajima and co-authors [13] found that reading and writing skills at age 12 positively predicted upper primary school completion by age 15 and upper secondary entry by age 19 among boys but not among girls. Marphatia and colleagues [19] reported that grade repetition increased the risk of discontinuation among boys but not among girls.

Drawing on data from a unique state-representative longitudinal study of adolescents aged 10–19 in the states of Bihar and Uttar Pradesh in India, this paper extends the literature on the relationship between learning deficits and early school leaving. Specifically, it examines the levels and patterns of early school leaving among adolescents, the relationship between learning deficits and early school leaving and the gender dimensions, if any, of this relationship.

## Materials and methods

### Study setting

The study (Understanding the lives of adolescents and young adults, hereafter referred to as UDAYA study) was conducted in the states of Bihar and Uttar Pradesh in India. Uttar Pradesh, with a projected population of 238.1 million in 2024, has the largest population of any state in the country, accounting for 17% of India’s population [22]. Bihar, with a projected population of 128.6 million, is the second largest state in terms of population, accounting for 9% of India’s population. Bihar and Uttar Pradesh, with a projected combined adolescent population of 72.9 million in 2021, are home to every third adolescent aged 10−19 in India. Both states are predominantly rural; population projection data show that 88% of the population in Bihar and 76% in Uttar Pradesh resided in rural areas in 2024. Economically, Bihar and Uttar Pradesh are the poorest among all the states and union territories in India with per capita income well below the national average and the lowest among the states – INR 54,111 in Bihar and INR 83,636 in Uttar Pradesh as against INR 169,496 nationally at current prices in 2022−23 [23].

Indicators of education participation and performance of children and adolescents highlight poor performance of both states, particularly, Bihar. While net enrolment rate was at par with the national rate at the primary school level (90% in Bihar, 87% in Uttar Pradesh and 91% nationally), rates at the lower secondary and upper secondary levels were well below the national average (35%, 34% and 48%, respectively, in Bihar, Uttar Pradesh and nationally, at the lower secondary level and 18%, 25% and 34%, respectively, at the upper secondary level in 2021–22; [24]). While the dropout rate at primary school level was low in both states (<5%), dropout rate for lower secondary school was 21% in Bihar and 10% in Uttar Pradesh in 2021–22. The Annual Status of Education Report surveys show that the proportion of children who could read grade 2 level text fluently among those who were enrolled in grade 8 declined from 81% in 2012 to 71% in 2022 in rural Bihar and stagnated at 70–71% in rural Uttar Pradesh [25]. While those who could solve a division problem declined from 67% to 60% in rural Bihar, the proportion increased from 37% to 49% in rural Uttar Pradesh during this period [25].

Education system data from both states indicate that gender gaps are negligible in most indicators related to school participation. However, significant gender gaps in learning levels are noticeable in both states, mostly favouring boys. For example, average score of grade 10 male students was higher than that of female students in modern Indian language (257 vs. 245 out of 500 in Bihar and 255 vs. 249 in Uttar Pradesh), mathematics (237 vs. 222 in Bihar and 216 vs. 207 in Uttar Pradesh), science (210 vs. 195 in Bihar and 194 vs. 188 in Uttar Pradesh), social science (229 vs. 216 in Bihar and 217 vs. 211 in Uttar Pradesh) and English (265 vs. 253 in Bihar and 256 vs. 250 in Uttar Pradesh) in 2021 [26].

### Study design

The UDAYA study from which data presented in this paper were drawn is the first-ever longitudinal study of a state-representative sample of adolescents aged 10–19 in Bihar and Uttar Pradesh (UDAYA data and tools are available at Harvard Dataverse, V2; [27,28]). The first wave of the survey was conducted in 2015–2016 and the second wave was conducted in 2018–2019. The goal of the study was to establish the levels, patterns and trends in the situation of younger (10–14) and older (15–19) adolescents and to assess factors that influence the quality of transitions they make. The study was designed to provide state-level estimates for five categories of adolescents, namely, unmarried boys aged 10–14, unmarried boys aged 15–19, unmarried girls aged 10–14, unmarried girls aged 15–19 and married girls aged 15–19. The study adopted a systematic, multi-stage stratified sampling design to draw primary sampling units independently for rural (75 villages) and urban (75 wards) areas. In each primary sampling unit, households to be interviewed were selected by systematic sampling (for more details about the study design, see [29]). A total of 20,594 adolescents were interviewed in the first wave, with a response rate of 92% and just 1% of selected respondents refused to participate. The main reason for non-response was that the respondent was not at home (5%). Of the 20,594 respondents who were eligible for re-interview, 16,818 adolescents were reinterviewed. Three percent of respondents who gave inconsistent response to questions related to age and education at the follow-up survey were excluded; therefore, the final follow-up sample comprised 16,292 respondents, thus resulting in an effective follow-up rate of 79%. The main reasons for loss-to-follow-up were that the participant had migrated and the participant or his/her parent or guardian refused (for more details about the follow-up survey, see [30]). The questionnaire and the tools used in wave 2 replicated those used in wave 1 in most respects to ensure comparability across waves.

Our descriptive analysis of early school leaving and learning deficits drew on data from adolescents who were ever enrolled in school and interviewed at both waves (N = 11,476, comprising 7,159 girls and 4,317 boys). Enrolment in school was nearly universal in the study sample (just 4% were never enrolled in school) and as such, the background characteristics of the sample of ever-enrolled adolescents did not differ with those of the overall UDAYA sample at wave 1 (see S1 Table). Multivariate analysis of the relationship between learning deficits and early school leaving drew on data from adolescents who were enrolled in school, including distance education courses, at wave 1 and interviewed at both waves (N = 9,169, comprising 5,493 girls and 3,676 boys). Thus, we were able to ensure that first round of learning assessment which took place along with the first wave of interview preceded the outcome variable of early school leaving. The background characteristics of the sample of currently enrolled adolescents differed with those of the overall UDAYA sample at wave 1 (See S1 Table). The currently enrolled adolescents were younger, better educated and wealthier than the overall UDAYA sample. Additionally, a larger proportion of the currently enrolled adolescents belonged to Hindu religion and socially privileged castes and resided in Bihar.

We note that 21% of the ever-enrolled adolescents and 19% of the currently enrolled adolescents were lost to follow up in the second wave of the UDAYA study (See S2 Table). We fitted probit regression model to examine the extent to which the socio-demographic characteristics of ever/currently enrolled adolescents who were re-interviewed at wave 2 and those who were not differed. We found that the characteristics of those who were re-interviewed and those who were not differed significantly in terms of years of schooling completed, religion, caste, household wealth, place of residence and state of residence (See S3 Table). Those who belonged to socially disadvantaged castes and tribes (i.e., scheduled castes and tribes and other backward castes), those from economically better-off households and those from Bihar were more likely than others to be re-interviewed. On the other hand, those who were better educated, Muslim and resident of urban areas were less likely than others to be re-interviewed. We controlled for these variables in the discrete-time hazard model and the fixed effects model, as appropriately (see S4 Table).

We excluded girls who were married at wave 1 from the analysis because only 11% of married girls were enrolled in school at wave 1. Moreover, married boys were not included in the UDAYA study because early marriage is not so common among boys, and thus, the exclusion of married girls’ sample made gender comparisons neater.

The Population Council’s Institutional Review Board approved the UDAYA study from which data were drawn for this paper (Protocol # 698). In the UDAYA study, written consent was sought from individuals to be interviewed, and for minors, consent was also sought from a parent or guardian at both wave 1 and wave 2. Moreover, all survey respondents at wave 1 were asked if they would consent to being re-interviewed three years later. In the case of minors, parents/guardians were also asked whether they would permit the research team to approach their daughter/son three years later for a re-interview. Only those who had provided consent to be re-interviewed at the time of the original interview at wave 1 were approached for re-interview at wave 2. Signing the consent form was optional to preserve the confidentiality of the respondent and/or the parent/guardian; however, the interviewer was required to sign a statement that she or he had explained the content of the consent form to the respondent or parent. No additional consent was taken for the analysis presented in this paper because we conducted secondary analysis of deidentified data and the data were analysed anonymously.

### Variables

#### Early school leaving.

We defined early school leaving, the outcome variable, in terms of discontinuing schooling before completing secondary education, that is, grade 12. We measured it in two ways: (1) cumulative probability of leaving school before completing grade 12 among ever enrolled adolescents aged 13–22 at wave 2; and (2) the proportion of ever enrolled young people aged 18–22 at wave 2 who discontinued schooling before completing grade 12. We note that the official entrance age to primary education is 6 years and theoretical duration for completing secondary education is 12 years in these states. Therefore, young people aged 18 and above are theoretically expected to have completed secondary education.

#### Learning deficits.

Our key explanatory variable is learning deficits. We defined learning deficits in terms of foundational skills. The UDAYA study used the Annual Status of Education Report (ASER) survey tools to assess literacy and numeracy levels among all survey participants who were ever enrolled in school; ASER tools have been used to measure foundational literacy and numeracy among children and adolescents in India for over two decades and have been observed to be reliable and valid. The literacy assessment tool assessed ability to read in the native language, Hindi and consisted of four levels: recognition of letters, ability to read words, ability to read a short paragraph (Grade 1 level text) and ability to read a longer ‘story’ (Grade 2 level text) [31]. Respondents were marked at the highest level which they could read comfortably, that is, with no more than three mistakes. The numeracy assessment tool consisted of four levels: recognition of single-digit numbers (1–9), recognition of double-digit numbers (11–99), two-digit subtraction sum with carry-over (Grade 2 level) and a three-digit division sum (Grades 3–4 level). Respondents were marked at the highest level which they could do correctly. We created a four-category variable of learning deficits, based on these assessments: (1) no learning deficit, that is, can read grade 2 text fluently and solve a division problem, (2) can read grade 2 text fluently, but cannot solve a division problem, (3) cannot read grade 2 text fluently, but can solve a division problem and (4) severe learning deficit, that is, cannot read grade 2 text or solve a division problem.

#### Other covariates.

Our multivariate analyses controlled for several individual, household/family and school related variables that are found to be associated with school discontinuation in earlier studies. These variables included age, sex, engagement in paid work, marital status, decision-making say in personal matters, freedom of movement, gender role attitudes and age at first-time enrolment in school (see S4 Table for a brief description of these variables). At the household/family level, variables controlled for included household wealth, religion, caste, mother’s education, parent-child communication on personal matters and gender discriminatory practices by parents. School-related variables that were controlled for included type of school attended, private coaching and enrolment in schools with basic amenities (such as drinking water, functional toilet, playground and library). We also controlled for place of residence and the state of residence. While discrete-time hazard models controlled for both time-varying and time-invariant covariates, fixed effects models controlled for time-varying covariates.

Table 1 shows the summary statistics at wave 1 and wave 2 for covariates other than learning deficits. Ever-enrolled study participants were aged on average 17 years at wave 2 and substantial proportions had engaged in paid work in the year preceding the interview (22–38% at wave 2). Some 11% of girls and 4% of boys got married during the inter-survey period. Having a say in personal matters increased over time and 62% of girls and 78% of boys reported some say at wave 2. Likewise, freedom of movement improved over time and 52% of girls and 93% of boys reported freedom to visit selected locations unescorted at wave 2. Most study participants expressed gender egalitarian attitudes and adherence to egalitarian attitudes increased over time. Most study participants were from rural areas (65–77%) and belonged to Hindu religion (77–86%). More than half of the respondents belonged to other backward castes (54–55%) and a quarter belonged to scheduled castes or tribes (23–27%). The mean household wealth index score was 25/26 out of 57 at wave 2. Mothers of most study participants had no formal education. Communication with parents was far from universal. Small proportions of study participants had acknowledged gender discriminatory practices at home, where parents favoured sons over daughters (8–13% at wave 1). Most study participants were enrolled in a government educational institution and availability of basic amenities—drinking water, a playground, toilets, and libraries—was limited in the schools in which they were enrolled. A notable proportion of study participants had received private tutoring in the month prior to the interview.

**Table 1:** Summary statistics for explanatory variables other than learning deficits used in the multivariate analyses, Bihar and Uttar Pradesh, 2015−16 and 2018−19.

	Ever enrolled				Currently enrolled at wave 1			
	Girls		Boys		Girls		Boys	
Variables	2015−16	2018−19	2015−16	2018−19	2015−16	2018−19	2015−16	2018−19
Age (Mean)	13.7 [2.6]	16.6 [2.6]	14.0 [2.7]	17.0 [2.8]	13.3 [2.4]	16.2 [2.5]	13.6 [2.6]	16.6 [2.7]
Engagement in paid work (%)	11.4	22.2	19.6	38.0	8.0	20.3	13.1	31.2
Got married during the inter-survey period (%)	0.0	10.6	0.0	4.4	0.0	7.3	0.0	2.5
Decision-making say (%)	42.6	61.8	56.2	78.3	45.3	62.0	56.1	76.7
Freedom of movement (%)	40.7	52.2	82.2	93.4	42.6	56.3	80.4	92.9
Gender role attitudes (%)	51.0	69.3	40.5	52.8	52.8	71.9	40.7	54.2
Age at first-time enrolment (Mean)	5.6 [1.4]	--	5.7 [1.3]	--				
Household wealth (Mean)	22.2 [9.2]	25.5 [8.4]	20.8 [8.8]	25.2 [7.9]	22.7 [9.4]	25.9 [8.5]	21.2 [8.9]	25.5 [7.9]
Religion (%)								
Hindu	77.1	**--**	85.5	--	80.3	--	86.4	--
Muslim and others	22.9	--	14.6	--	19.7	--	13.6	--
Caste (%)								
Scheduled caste/tribe	23.3	--	27.0	--	22.5	--	26.0	--
Other backward caste	54.2	--	55.3	--	54.0	--	55.4	--
General caste	22.5	--	17.7	--	23.5	--	18.6	--
Mother’s education – literate (%)	35.0	--	30.2	--	38.6	--	31.4	--
Place of residence – Rural (%)	67.6	65.4	83.5	77.3	67.3	64.6	83.7	77.5
Parent-child communication (Mean)	1.8 [1.0]	1.7 [1.1]	1.2 [0.8]	1.1 [0.9]	2.0 [0.9]	1.9 [1.1]	1.4 [0.8]	1.2 [0.9]
Gender discriminatory practices at home (%)	12.5	9.1	7.6	11.0	12.0	9.0	7.7	11.6
Attended private school (%)	38.5	37.2	44.0	43.8	40.8	39.2	45.7	45.3
Received private coaching (%)	28.4	27.2	38.0	36.9	33.7	32.1	43.5	42.1
Attended schools with basic amenities (%)	36.9	49.4	26.6	39.5	38.0	52.5	28.2	42.9
State of residence – Bihar (%)	27.3	--	33.0	--	29.2	--	34.4	--
Number of respondents	7,159	7,159	4,317	4,317	5,493	5,493	3,676	3,676

Values in the parentheses show standard deviation.

### Analysis

We used life table analysis to examine the cumulative probability of early school leaving among those who were ever enrolled in school, disaggregated by background characteristics. We used life table analysis because those who have not experienced school leaving by wave 2 or earlier but who are still at risk of the event can be included in the life table analysis. Dropout was the failure event and those who had continued schooling by wave 2 were censored. We applied the log rank test to test the null hypothesis of no difference in early school leaving between different sub-groups. We calculated the proportion of young people who discontinued schooling before completing grade 12 among those who were ever enrolled and were aged 18 and above at wave 2 to complement the findings of the life table analysis.

We fitted discrete-time hazard model using logistic regression to examine the relationship between learning deficits and early school leaving. The advantage of the discrete-time model over various continuous models is that it makes no assumption about the shape of the hazard-rate function [14]. The period of observation started at the grade that respondents attended at the time of wave 1 interview to ensure that the wave 1 assessment of learning deficits preceded the occurrence of early school leaving. We used information reported in two survey waves, with information that changed over time being considered as time-varying covariates. Unfortunately, we did not have information on the exact timing of a change in several time-varying covariates (e.g., respondents’ learning deficits, decision-making say, freedom of movement, gender role attitudes, household wealth, parent-child communication, gender discriminatory experiences at home, type of school attended, private coaching and enrolment in schools with basic amenities). We used data from wave 1 for the first half of person-years of exposure and data from wave 2 for the second half of person-years of exposure for such variables.

Examining the association between learning deficits and early school leaving is complicated by the issue of endogeneity arising from simultaneity, omitted variables and measurement errors. Therefore, we also used fixed effects regression model to examine the relationship between learning deficits and early school leaving. We opted for fixed effects regression because it leverages the longitudinal structure of the data and estimates within-subject difference in the outcome variable over time as a function of within-subject difference in explanatory variables. It also addresses endogeneity by controlling for time-invariant characteristics of the respondents or their environments and eliminates omitted variable bias [32]. The Hausman test results confirmed that fixed effects model was more appropriate than the random effects model for our analysis. The fixed effects regression analysis drew on the subsample of adolescents who were enrolled in grades below 12 at wave 1 and were aged 18 and above at wave 2.

We fitted separate models for all adolescents and for girls and boys separately to capture differences in the effect of learning deficits on early school leaving between girls and boys. We applied Z test to examine whether the regression coefficients from the girls’ and boys’ models differed significantly. All analyses were conducted using STATA software (version 16.1).

## Results

### Early school leaving

**Fig 1:**
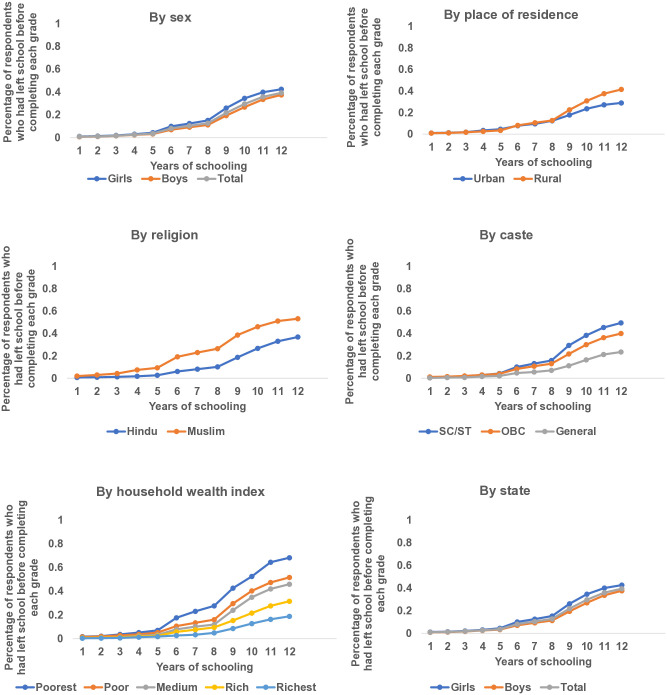
Cumulative probability of young people aged 13−22 years leaving school before completing each grade by selected chacteristics, Bihar and Uttar Pradesh, 2018−19.

Findings indicate that early school leaving was substantial among adolescents in Bihar and Uttar Pradesh and that the probability of early school leaving differed significantly by adolescents’ background characteristics (Fig 1). Although discontinuation was minimal before completing grades 1–5 (1–4%), dropout rate rose steadily thereafter, reaching 8–13% before completing grades 6–8, 22–30% before completing grades 9–10 and 39% before completing grade 12. While gender gap in school discontinuation was negligible or at best modest before completing grade 8, the gap widened thereafter, with a higher dropout rate among girls than boys, for example, 42% versus 38% dropout rate before completing grade 12. Rural-urban gap in school discontinuation was, likewise, at best modest before completing grade 8, while the gap widened thereafter, with a higher dropout rate before completing grade 12 among rural than urban adolescents (42% vs. 29%). Adolescents belonging to Muslim religion had a higher probability of early school leaving than those belonging to Hindu religion; dropout rate was noticeable even before completing grade 4 among the former, with dropout rate rising steadily thereafter to reach 53% before completing grade 12 compared to 37% among the latter. Caste-wise difference was also substantial, with the highest rate of early school leaving found among adolescents belonging to scheduled castes and tribes (50%), followed by those belonging to other backward castes (40%) and general castes (24%). The probability of early school leaving steadily declined with household wealth, from 68% among adolescents belonging to the poorest households to 19% among those belonging to the richest households. While the rate of school leaving was similar in Bihar and Uttar Pradesh until before completing grade 9, the rate diverged thereafter, with Bihar reporting a higher rate of school leaving before completing grade 12 than Uttar Pradesh (45% vs 36%).

**Fig 2:**
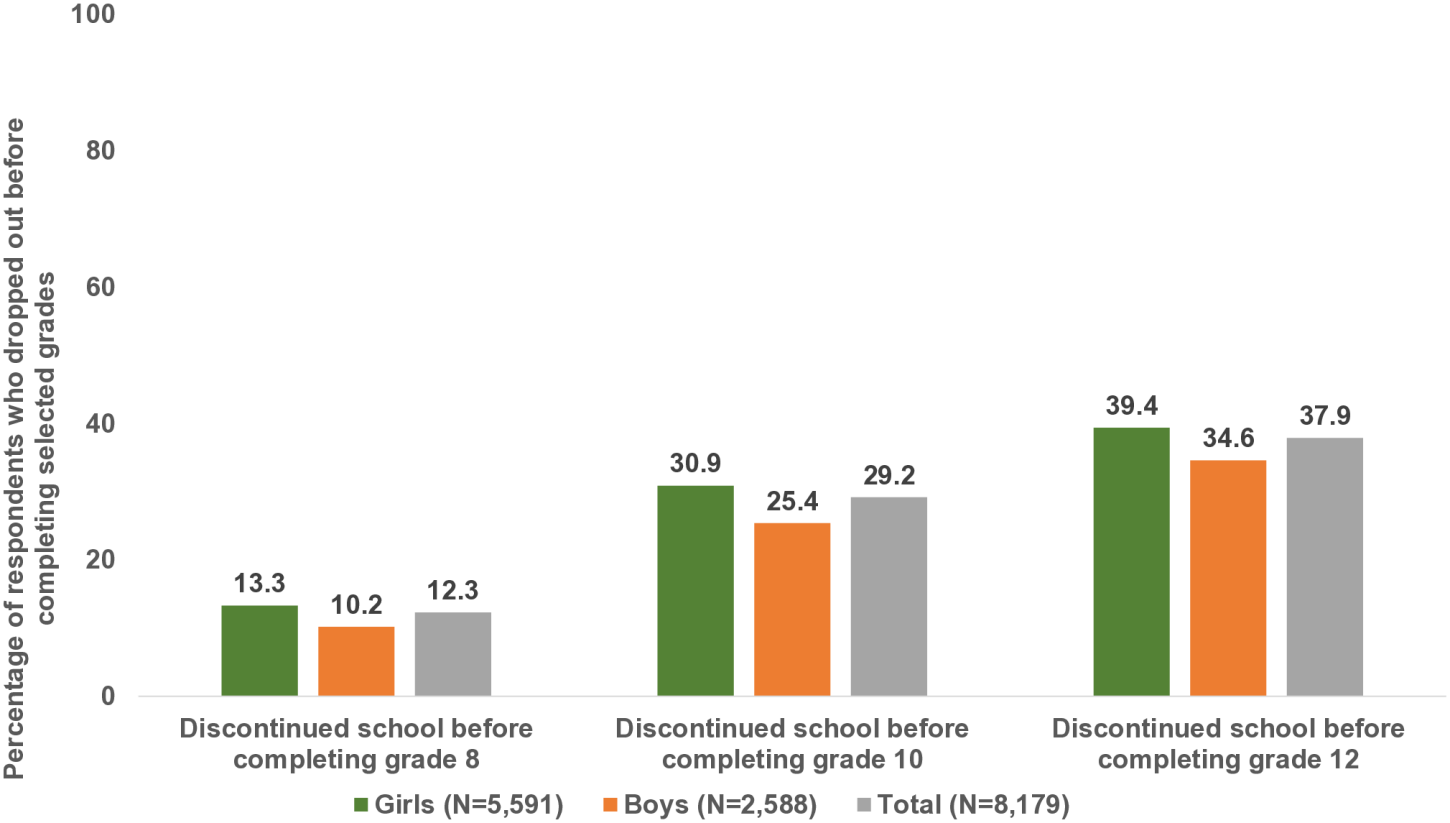
Percentage of young people aged 18−22 years who dropped out before completing selected grades by sex, Bihar and Uttar Pradesh, 2018−19.

Estimates of early school leaving obtained using data from those who would have theoretically transitioned out of school, that is, those who were ever enrolled and were aged 18 and above at wave 2, were similar to those obtained by the life table analysis (Fig 2). The level of early school leaving rose steadily from 12% before completing grade 8 to 29% before completing grade 10 and 38% before completing grade 12. Moreover, a larger proportion of girls than boys left school before completing each of these milestones.

### Learning deficits

Most adolescents experienced learning deficits of varying intensity. Only 41% of adolescents aged 10–19 were able to read a grade 2 text fluently in Hindi and solve a simple division problem at wave 1, with boys scoring better than girls (46% vs. 29%; Table 2). At the other extreme, 25% of adolescents displayed severe learning deficit, that is, they could not read grade 2 text or solve a simple division problem, with a slightly more girls than boys falling into this group (29% vs. 23%). Population-based analysis shows that learning levels improved over time – the percentage of adolescents who could read a grade 2 text fluently and solve a division problem increased to 47% at wave 2; this improvement was observed among both girls (35%) and boys (53%).

**Table 2:** Percentage distribution of young people who were ever enrolled in school by learning levels, Bihar and Uttar Pradesh, 2015−16 and 2018−19.

Learning levels (%)	2015−16			2018−19		
	Girls (10–19 years)	Boys (10–19 years)	Total (10–19 years)	Girls (13–22 years)	Boys (13–22 years)	Total (13–22 years)
Can read grade 2 text and solve a division problem	29.4	45.6	41.0	35.3	53.2	47.3
Can read grade 2 text, but cannot solve a division problem	41.1	30.2	32.5	45.8	28.3	33.1
Cannot read grade 2 text, but can solve a division problem	0.7	1.6	1.4	0.4	1.8	1.4
Cannot read grade 2 text or solve a division problem	28.9	22.6	25.1	18.6	16.8	18.2
Number of respondents ever enrolled and took learning assessment	8,796	5,766	14,562	6,743	4,181	10,924

**Fig 3:**
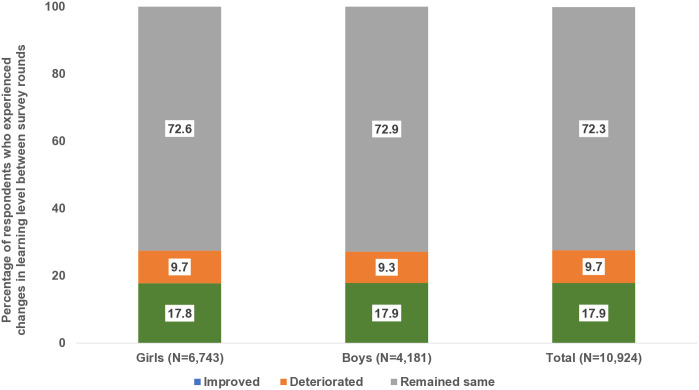
Percentage of young people aged 13−22 years, according to changes in learning levels experienced between survey rounds, Bihar and Uttar Pradesh, 2015−16 and 2018−19.

Cohort-wise analysis shows that improvement in learning levels was not linear (Fig 3). While learning levels improved for 18% of adolescents, levels declined for 10% and remained unchanged for 73% between the two waves of data collection. Similar levels of changes were observed for girls and boys.

### Relationship between learning deficits and early school leaving

Results from the discrete-time hazard models presented in Table 3 show that learning deficits influenced the probability of early school leaving and that there was a dose effect, with the effect size three times as large for those who could not read grade 2 text fluently and solve a division problem (β = 1.959, p < 0.001) than those who could read grade 2 text fluently but could not solve a division problem (β = 0.568, p < 0.001). Learning deficits affected the probability of early school leaving among girls and boys (β = 0.372, p < 0.05 and β = 0.705, p < 0.001, respectively, for those with moderate learning deficits; β = 1.822 and 2.057, p < 0.001, respectively, for those with severe learning deficits). A comparison of regression coefficients, using Z test, shows that coefficients for girls and boys did not differ significantly (z = 1.3592, p = 0.174 for moderate learning deficits and z = 0.77388, p = 0.439 for severe learning deficits).

**Table 3:** Effect of learning deficits on early school leaving, according to discrete-time hazard models.

Learning deficits indicators	Girls (β coefficient)	Boys (β coefficient)	Total (β coefficient)
Moderate learning deficit	0.372 [0.042; 0.014–0.729]	0.705 [0.000; 0.384–1.027]	0.568 [0.000; 0.316–0.821]
Severe learning deficit	1.822 [0.000; 1.359–2.285]	2.057 [0.000; 1.683–2.432]	1.959 [0.000; 1.669–2.249]
Number of person-years	14,352	10,775	25,127
Wald chi2(17)	563.29	552.62	921.07
Prob>chi2	0.000	0.000	0.000
Pseudo R^2^	0.2841	0.2954	0.2855
Log pseudolikelihood	−3699.0481	−1618.1391	−4756.1021
Number of respondents^@^	5,304	3,580	8,884

@ We excluded 285 respondents who did not attempt the learning assessment at wave 2. Values in the parentheses refer to p value and 95% confidence intervals.

Although not exactly comparable, results of the fixed effects regression reiterate the relationship between learning deficits and early school leaving (Table 4). Findings show that severe learning deficits influenced early school leaving (β = 0.195, p < 0.05). However, no such relationship was observed for moderate learning deficits. Findings from the gender disaggregated fixed effects analysis were consistent with those of the discrete-time hazard analysis. Severe learning deficits were associated with early school discontinuation for girls and boys (β = 0.155, p < 0.001 and β = 0.213, p < 0.05, respectively). Here again, a comparison of regression coefficients shows that coefficients for girls and boys did not differ significantly (z = −0.5065, p = 0.613).

**Table 4:** Effect of learning deficits on early school leaving, according to fixed effects models.

Learning deficits indicators	Girls (β coefficient)	Boys (β coefficient)	Total (β coefficient)
Moderate learning deficit	−0.004 [0.805; −0.040–0.031]	0.033 [0.270; −0.025–0.090]	0.024 [0.286; −0.020–0.069]
Severe learning deficit	0.155 [0.001; 0.063–0.247]	0.213 [0.042; 0.008–0.417]	0.195 [0.022; 0.028–0.362]
F-stat	34.20***	14.57***	31.34***
F-test for individual effects	0.97	0.85	0.95
Rho	0.367	0.317	0.343
Hausman test: Chi-square	378.7	109.4	510.8
Number of respondents	3,044	1,579	4,623

Values in the parentheses refer to p value and 95% confidence intervals.

### Factors other than learning deficits affecting early school leaving

Results from the discrete-time hazard model presented in Table 5 show that the probability of early school leaving increased with age (β = 0.423, p < 0.001). Compared to girls, the probability of early school leaving was lower for boys (β = −0.398, p < 0.01). The probability of early school leaving was higher among adolescents who were engaged in paid work (β = 1.281, p < 0.001) and who got married during the inter-survey period (β = 1.180, p < 0.001). Adolescents who held gender egalitarian attitudes had lower probability of early school leaving than others (β = −0.502, p < 0.001). Indicators related to adolescents’ agency such as decision-making say and freedom of movement were found to be not related to the probability of early school leaving in the discrete-time hazard models. The probability of early school leaving declined with age at first-time enrolment in school which was counter-intuitive (β = −0.181, p < 0.001). Adolescents who were Muslims had higher probability of early school leaving than Hindus (β = 0.813, p < 0.001). On the other hand, adolescents who belonged to wealthier households had lower probability of early school leaving (β = −0.054, p < 0.01). So did adolescents whose mother was literate (β = −0.428, p < 0.001) and whose parents discussed personal matters with them (β = −0.218, p < 0.001). The probability of early school leaving was lower for adolescents who attended schools with basic amenities (β = −0.591, p < 0.001) and who were enrolled in private schools (β = −0.323, p < 0.001) than others. Finally, adolescents who resided in Bihar had higher probability of early school leaving than those from Uttar Pradesh (β = 0.434, p < 0.001).

**Table 5:** Estimated effect of explanatory variables other than learning deficits on early school leaving, according to discrete-time hazard models.

Variables	Girls (β coefficient)	Boys (β coefficient)	Total (β coefficient)
Age	0.395*** [0.328–0.462]	0.406*** [0.335–0.478]	0.423*** [0.374–0.472]
Boys (Ref. Girls)			−0.398** [−0.655 – −0.141]
Engagement in paid work (Ref. No)	1.000*** [0.603–1.398]	1.601*** [1.316–1.886]	1.281*** [1.066–1.497]
Got married during the inter-survey period (Ref. No)	1.518*** [1.084–1.953]	0.261 [−0.373–0.896]	1.180*** [0.821–1.538]
Age at first-time enrolment	−0.081 [−0.201–0.038]	−0.208*** [−0.324 – −0.092]	−0.181*** [−0.264 – −0.099]
Decision-making say	0.169 [−0.127–0.465]	−0.356* [−0.641 – −0.071]	−0.148 [−0.359–0.063]
Freedom of movement (Ref. No)	−0.093 [−0.393–0.207]	−0.157 [−0.596–0.281]	−0.027 [−0.278–0.225]
Gender role attitudes	−0.407* [−0.728 – −0.086]	−0.607*** [−0.934 – −0.280]	−0.502*** [−0.728 – −0.275]
Household wealth	−0.069*** [−0.095 – −0.009]	−0.036*** [−0.058 – −0.015]	−0.054*** [−0.070 – −0.037]
Muslim and others (Ref. Hindu)	0.798*** [0.394–1.203]	0.780*** [0.332–1.227]	0.813*** [0.498–1.128]
Other backward caste (Ref. Scheduled caste/tribe)	−0.299 [−0.662–0.063]	−0.183 [−0.519–0.153]	−0.179 [−0.420–0.063]
General caste	−0.106 [−0.595–0.384]	0.001 [−0.497–0.498)]	−0.021 [−0.388–0.347]
Literate mothers (Ref. Illiterate)	−0.547** [−0.938 – −0.156]	−0.317 [−0.643–0.008]	−0.428*** [−0.678 – −0.179]
Urban residence (Ref. Rural)	−0.072 [−0.427–0.282]	0.143 [−0.197–0.483]	0.040 [−0.211–0.292]
Parent-child communication (Ref. No)	−0.172** [−0.302 – −0.041]	−0.298*** [−0.477 – −0.118]	−0.218*** [−0.326 – −0.109]
Gender discriminatory practices at home (Ref. No)	0.169 [−0.239–0.578]	0.298 [−0.087–0.683]	0. 227 [−0.056–0.510]
Enrolment in private school (Ref. Government school)	−0.272 [−0.641–0.097]	−0.225 [−0.529–0.079]	−0.323** [−0.564 – −0.081]
Private coaching (Ref. No)	−0.474*** [−0.759 – −0.188]	−0.099 [−0.405–0.207]	−0.206 [−0.435–0.022]
Enrolment in schools with basic amenities (Ref. No)	−0.424* [−0.756 – −0.092]	−0.640*** [−0.948 – −0.332]	−0.591*** [−0.818 – −0.365]
Bihar (Ref. Uttar Pradesh)	0.203 [−0.099–0.506]	0.465* [0.312–0.945]	0.434*** [0.200–0.668]
Number of person-years	14,352	10,775	25,127
Wald chi2(17)	563.29	552.62	921.07
Prob>chi2	0.000	0.000	0.000
Pseudo R^2^	0.2841	0.2954	0.2855
Log pseudolikelihood	−3699.0481	−1618.1391	−4756.1021

Note: *p<=0.05. **p<=0.01. ***p<=0.001; Values in the parentheses refer to p value and 95% confidence intervals.

While some factors affected the probability of early school leaving equally among girls and boys (for example, age, gender role attitudes, household wealth, religion, parent-child communication and enrolment in schools with basic amenities), other factors had gender differentiated effect. For example, engagement in paid work had greater effect on the probability of early school leaving for boys than girls (β = 1.601, p < 0.001 vs. β = 1.000, p < 0.001). Several others – marriage during the inter-survey period, mother’s education, private coaching – predicted the probability for girls but not for boys. And age at first-time enrolment in school, decision-making say and residence in Bihar predicted the probability for boys but not for girls.

Results of the fixed effects models reiterate that age, engagement in paid work, marriage during the inter-survey period, parent-child communication, enrolment in private school and enrolment in schools with basic amenities affected the chances of early school leaving (Table 6). Fixed effects regression models additionally show that adolescents who exercised some say in personal matters and who received private coaching had higher chances of early school leaving. However, factors such as adherence to gender egalitarian attitudes and household wealth that were found to influence early school leaving in the discrete-time hazard models were not found to be associated with it in the fixed effects models. While most of these factors equally affected the probability of early school discontinuation among girls and boys, some factors predicted the probability of early school leaving only for girls (marriage during the inter-survey period increased and freedom of movement decreased the probability), while others predicted the probability of early school leaving only for boys (engagement in paid work increased and enrolment in private school decreased the probability).

**Table 6:** Estimated effect of explanatory variables other than learning deficits on early school leaving, according to fixed effects models.

Variables	Girls (β coefficient)	Boys (β coefficient)	Total (β coefficient)
Age	0.050*** [0.042–0.059]	0.054*** [0.042–0.067]	0.057*** [0.047–0.066]
Engagement in paid work (Ref. No)	−0.005 [−0.039–0.029]	0.120*** [0.068–0.173]	0.083*** [0.040–0.126]
Got married during the inter-survey period (Ref. No)	0.161*** [0.076–0.245]	0.028 [−0.109–0.165]	0.123** [0.041–0.204]
Decision-making say	0.046*** [0.021–0.071]	0.053* [0.011–0.094]	0.053*** [0.022–0.084]
Freedom of movement (Ref. No)	−0.032* [−0.059 – −0.004]	−0.052 [−0.203–0.099]	−0.042 [−0.097–0.014]
Gender role attitudes	−0.012 [−0.047–0.024]	0.006 [−0.043–0.056]	0.002 [−0.036–0.040]
Household wealth	−0.001 [−0.004–0.002]	−0.001 [−0.004–0.003]	−0.001 [−0.003–0.002]
Urban residence (Ref. Rural)	−0.029 [−0.099–0.041]	0.013 [−0.087–0.112]	0.007 [−0.075–0.089]
Parent-child communication (Ref. No)	−0.072*** [−0.090 – −0.054]	−0.057*** [−0.082 – −0.031]	−0.068*** [−0.087 – −0.049]
Gender discriminatory practices at home (Ref. No)	0.023 [−0.006–0.052]	−0.010 [−0.056–0.037]	0.004 [−0.029–0.036]
Enrolment in private school (Ref. Government school)	0.006 [0.091–0.134]	−0.050*** [−0.080 – −0.020]	−0.050*** [−0.077 – −0.024]
Private coaching (Ref. No)	0.113*** [0.006–0.052]	0.079*** [0.046–0.112]	0.092*** [0.064–0.119]
Enrolment in schools with basic amenities (Ref. No)	−0.080*** [−0.106 – −0.054]	−0.099*** [−0.137 – −0.061]	−0.098*** [−0.128 – −0.068]
F-stat	34.20***	14.57***	31.34***
F-test for individual effects	0.97	0.85	0.95
Rho	0.367	0.317	0.343
Hausman test: Chi-square	378.7	109.4	510.8
Number of respondents	3,044	1,579	4,623

Note: *p<=0.05. **p<=0.01. ***p<=0.001; Values in the parentheses refer to p value and 95% confidence intervals.

## Discussion

The economic, social and health costs of not completing a full cycle of basic education and not acquiring basic skills are steep. Although the body of evidence on the relationship between foundational skills deficits and early school leaving has expanded, there is a need for additional rigorous research to shed light on the causal relationship between the two, particularly in settings in which girls and boys are at high risk of learning poverty and discontinuation. Our study responds to this need and provides rigorous evidence on the relationship between learning deficits and early school leaving among a longitudinal cohort of adolescents in India’s two most populous states. Our study also provides robust evidence on factors other than learning deficits that affected early school leaving.

Although education system data have shown modest levels of school discontinuation at secondary level (13% nationally and 14% in Bihar and Uttar Pradesh; [24]), our study indicates that early school leaving was substantially high among adolescents in Bihar and Uttar Pradesh. Moreover, early school leaving was higher for adolescents belonging to socio-economically disadvantaged groups than others. Our findings mirror those from community-based sample surveys in India [33–35].

Learning deficits of varying intensity affected most adolescents in Bihar and Uttar Pradesh. These findings concur with observations from other surveys of learning levels in these states [25,26]. Our study also indicates that notable proportions of girls and boys did not retain their foundational skills over time, as observed in other longitudinal studies [36].

Contrary to education system data that have shown no gender difference in discontinuation rates, our study shows that a larger proportion of girls than boys left school before completing educational milestones such as completing grades 8, 10 and 12. This concurs with a secondary analysis of data from the 2017–18 nation-wide survey on education by the National Statistical Office which found that early school leaving was somewhat higher among females (76%) than males (72%) [34]. Our findings also show that girls performed worse than boys, particularly in numeracy. Gendered division of labour and gender discriminatory practices within the household, including greater demands on girls’ than boys’ time for performing household chores, decisions and investments favouring education of boys more than girls may affect disproportionately girls’ attendance and performance in school [15]. Gendered school and classroom dynamics, gender discrimination in access to formal employment and earnings and gender norms that condone early marriage of girls and confinement of women and girls to domestic spheres, similarly, can disincentivize girls’ school attendance and performance [14–16].

A functional level of literacy and numeracy is indispensable for children to learn other subjects, develop other higher-order cognitive skills such as problem solving, logical reasoning and critical thinking and achieve their full potential [37,38]. Several previous studies from India and elsewhere have acknowledged the role of basic foundational skills in school completion [6,11,13,18,20]. Our study reiterates this view; learning deficits affected early school leaving even after controlling for a range of individual, household and school level variables. Moreover, effect was larger for those with severe than moderate learning deficits. To the extent that success begets success, it may be that better learning outcomes improve goals and attitudes about continuing in school and increase the self-confidence to do what is in their control and needed to stay in school. It is also possible that better performing students elicit positive reinforcement and support from family, school and community to realise their educational aspirations.

Although both early school leaving and learning deficits were gendered among adolescents in the study – girls were more likely to leave school early and girls had greater learning deficits than boys – there was no gender dimension to the effect of learning deficits on early school leaving, highlighting that acquiring foundational skills are critical for educational progress of both girls and boys. Our findings, which show that learning deficits increased school leaving for both girls and boys, contrast with findings from an earlier study from India that reported that reading and writing skills at age 12 positively predicted upper primary school completion by age 15 and upper secondary entry by age 19 among boys but not among girls [13].

Several findings from our study showcase that gendered beliefs and practices influenced adolescents’, particularly girls’, chances of early school leaving. First, the discrete-time hazard model (but not the fixed effects model) shows that the probability of early school leaving was lower for girls and boys who held gender egalitarian attitudes than others. Second, gender-disaggregated fixed effects analysis shows that girls with freedom of movement were less likely to have discontinued schooling before completing grade 12 than girls who were not allowed to move freely. Third, findings from both discrete-time hazard model and the fixed effects model highlight the detrimental effect of marriage in adolescence on school completion for girls. Girls who got married during the inter-survey period had higher probability of leaving school early than those who remained unmarried. No such relationship was observed for boys among whom marriage is not as common. We note that 11% of girls and 1% of boys reported that they had discontinued their schooling during the inter-survey period because they got engaged or married. The association between these gendered practices and school participation is documented in several studies from South Asia and sub-Saharan Africa [39–48]. There are several pathways through which these gendered beliefs and practices may constrain adolescents’, particularly girls’, school completion. For example, adherence to egalitarian gender role attitudes can promote school participation and a sense of school belonging [49,50]. Moreover, some have found that adolescents with egalitarian attitudes are more likely to hold higher educational aspirations than others [51]. Likewise, mobility can reduce distance cost of attending school, give girls a public identity, increase their exposure to information and help them develop interpersonal skills and self-confidence. Early marriage can constrain girls’ school continuation because of new responsibilities that require girls to care for a new household, childbirth and childcare and limited capability of girls who marry young to assert themselves [40,52].

Findings show that not all dimensions of adolescents’ agency affected early school leaving in the same direction. As described above, adherence to egalitarian gender role attitudes reduced early school leaving among girls and boys. However, the fixed effects analysis (not in the discrete-time hazard model) shows that girls and boys with some say in decisions on personal matters had higher probability of early school leaving than others.

Transitioning into adult roles interfered with the chances of early school leaving. As with early marriage for girls, engagement in paid work increased the risk of early school leaving among adolescents, more so among boys than girls. The association between adolescents’ employment and school participation concurs with several other studies [6,7,9,11,18,48]. A mixed-method study in India found that combining schooling and work affects school participation and performance because of irregular attendance in school, inability to handle the pressures of both studying and working at a young age, not having time for school assignments at home and work-related distractions and physical exhaustion, and that those children who combined schooling and work gradually drop out of school [53].

The role of family/parental factors in adolescents’ school participation is widely acknowledged in the extant literature [6–9,11,18,21,48]. Parents can provide a richer home environment, access to better schools and supplemental learning opportunities, improve the cognitive development of children and influence children’s motivation and educational aspirations and achievement [6]. Our findings also highlight that several dimensions of parental/family resources influenced adolescents’ probability of early school leaving. The discrete-time hazard model shows that the probability of early school leaving declined with household wealth for girls and boys. It also shows that the probability of early school leaving was lower for girls whose mother was literate than girls whose mother was illiterate, although no such relationship was observed for boys. Finally, the discrete-time hazard model and the fixed effects model show that adolescents who reported that they discussed personal matters with their parent/s had lower chances of early school leaving than others.

Findings also show that adolescents who attended schools with basic amenities had lower likelihood of early school leaving than others. A review of literature on how school infrastructure affects children’s learning outcomes notes that there is strong evidence that the provision of and access to basic services increase the chances of pupils and teachers attending school, remaining healthy at school, and, in the case of teachers, staying in their profession [54]. Moreover, adolescents who attended a private school had lower chances of early school leaving than those who attended a government school; this finding corroborates the findings of an earlier study in India [21].

Findings from our analysis should be interpreted with some limitations in mind. First, we acknowledge that data presented in this paper came from just two states of India and our findings cannot be generalised to other states given the diversity within India in terms of the social, economic and demographic characteristics of the population, educational access and gender and social norms underlying school participation. Second, although the UDAYA overall sample at wave 1 was representative of adolescents aged 10–19 in Bihar and Uttar Pradesh, our analysis focused on adolescents ever/currently enrolled in school at wave 1 and there was some attrition bias between survey rounds. We applied sample weights recalculated in wave 2 that adjusted for non-random attrition, based on characteristics observed at wave 1, to minimize bias. We also controlled for characteristics that differed significantly between those who were re-interviewed and those who were not at wave 2 in the discrete-time hazard model and the fixed effects model, as appropriately. Third, the ASER tools that we used to measure learning levels, although reliable and valid [55], measured only whether adolescents had attained basic foundational literacy and numeracy. We acknowledge that beyond foundational skills, higher-order cognitive skills such as problem solving, logical reasoning and critical thinking as well as socioemotional skills may influence school discontinuation. The potential effect of these skills could not be explored for lack of data in the UDAYA study. Fourth, learning deficits and other time-varying covariates were measured at just two points over the three-year inter-survey period. We note that temporal changes in these variables may have been more precise had there been yearly or more frequent assessments and that the study may not have captured sudden or unexpected changes occurring between survey rounds, if any, in the time-varying covariates. Finally, our multi-variate analyses controlled for several individual, family and school-related co-variates; however, we acknowledge that there may be unmeasured co-variates that may have influenced outcomes.

Despite these limitations, our study makes major contributions to the literature on learning deficits and early school leaving. Our study highlights the need for concerted efforts to improve learning outcomes. Indeed, the pursuit of universal foundational literacy and numeracy in primary schools is a priority goal of India’s National Education Policy 2020 [56]. The Government of India has launched the National Initiative for Proficiency in Reading with Understanding and Numeracy (NIPUN Bharat) to achieve this goal [57]. Several measures have been proposed – child centred pedagogy, specific teacher training modules focusing on foundational literacy and numeracy designed through the National Initiative for School Heads’ and Teachers’ Holistic Advancement (NISHTHA, a teacher capacity building programme) and use of Digital Infrastructure for Knowledge Sharing (DIKSHA, a national digital platform for school education), for example. A strong commitment is needed to ensure that these measures are effectively implemented and that they reach the most disadvantaged groups.

A review of studies that used experimental or quasi-experimental designs to evaluate the impact of educational and related interventions on schooling outcomes in India shows that strategies such as awareness-building programmes for parents, increasing numbers of teachers, making improvements to pedagogy and instruction, remedial education, incentives to teachers and community- or school-based monitoring hold promises for improving learning outcomes [58]. For example, cluster randomized trials have shown positive effect of parental engagement activities along with remedial education by community volunteers and awareness-building programmes to inform parents on the social and economic gains of girls’ secondary education on learning outcomes [59–61]. Likewise, several trials have showcased the effectiveness of remedial education and improvements in pedagogy and lessons delivery (e.g., teaching students according to their ability level, delivery of a child-centric curriculum, structured pedagogy) on improving learning outcomes [59,61–67]. Strategies focused on teachers, including performance-based payments and incentives to teachers and hiring additional teachers as well as engaging community members and school management committees and school-based monitoring were also found to be promising [60,61,68–70]. Investments are required to adapt, re-evaluate, and upscale these promising models.

While investment in improving foundational skills is paramount for preventing early school leaving, they are not the only drivers of truncated education. Accompanying measures which address gendered beliefs and practices and premature transition to adult roles, enhance parental engagement and improve education systems, including education infrastructure, are also needed, with additional efforts targeting the most disadvantaged groups.

## Supporting information

S1 Table Percentage distribution of overall UDAYA sample and the analytical samples, according to selected background characteristics, Bihar and Uttar Pradesh, 2015−16. (DOCX)

S2 Table Number of respondents who were interviewed and who took learning assessments at wave 1 and wave 2, Bihar and Uttar Pradesh, 2015−16 and 2018−19. (DOCX)

S3 Table Results from the probit regression analysis of attrition bias. (DOCX)

S4 Table Description for explanatory variables used in the multivariate analyses. (DOCX)
