# Supplementary material for: Learning deficits and early school leaving: Evidence from a longitudinal study in India
Source: PLoS One. 2025 Nov 18;20(11):e0336850. doi: 10.1371/journal.pone.0336850 (PMC12626265; doi:10.1371/journal.pone.0336850)
Supplement: S1 Table — (DOCX) [file pone.0336850.s001.docx]

**S1 Table: Percentage distribution of overall UDAYA sample and the analytical samples, according to selected background characteristics, Bihar and Uttar Pradesh, 2015-16**

| **Selected characteristics (%)** | **All respondents aged 10-19 at wave 1** | | | **Respondents ever enrolled in school at wave 1** | | | **Respondents currently enrolled in school at wave 1** | | |
| --- | --- | --- | --- | --- | --- | --- | --- | --- | --- |
|  | **Girls** | **Boys** | **Total** | **Girls** | **Boys** | **Total** | **Girls** | **Boys** | **Total** |
| Age |  |  |  |  |  |  | *** | *** | *** |
| 10-14 | 17.6 | 34.9 | 24.3 | 18.0 | 35.4 | 24.9 | 23.3 | 41.0 | 30.9 |
| 15-19 | 82.5 | 65.1 | 75.7 | 82.0 | 64.6 | 75.1 | 76.7 | 59.0 | 69.1 |
| Place of residence |  |  |  |  |  |  |  |  |  |
| Urban | 20.8 | 15.1 | 17.3 | 20.8 | 14.8 | 17.0 | 20.8 | 14.7 | 16.9 |
| Rural | 79.2 | 84.9 | 82.7 | 79.2 | 85.2 | 83.0 | 79.2 | 85.3 | 83.1 |
| Religion |  |  |  |  |  |  | *** | *** | *** |
| Hindu | 77.9 | 84.1 | 81.7 | 79.4 | 85.0 | 82.9 | 82.1 | 86.0 | 84.6 |
| Muslim/Others | 22.1 | 16.0 | 18.3 | 20.6 | 15.0 | 17.1 | 17.9 | 14.0 | 15.4 |
| Caste |  |  |  |  |  |  | ** | ** | *** |
| Scheduled castes/tribes | 22.9 | 26.6 | 25.2 | 22.6 | 26.5 | 25.1 | 21.6 | 25.4 | 24.0 |
| Other backward castes | 58.3 | 55.7 | 56.6 | 57.9 | 55.7 | 56.5 | 58.2 | 55.8 | 56.7 |
| General castes | 18.9 | 17.7 | 18.2 | 19.5 | 17.8 | 18.4 | 20.2 | 18.8 | 19.3 |
| Years of schooling completed |  |  |  |  |  |  | *** | *** | *** |
| None | 5.6 | 2.7 | 3.8 | --- | --- | --- | --- | --- | --- |
| 1-4 years | 20.4 | 20.8 | 20.8 | 21.6 | 21.4 | 21.5 | 22.1 | 21.6 | 21.7 |
| 5-9 years | 58.0 | 59.3 | 59.3 | 61.5 | 61.0 | 61.2 | 60.8 | 60.0 | 60.3 |
| 10+ years | 16.0 | 17.1 | 17.1 | 16.9 | 17.6 | 17.4 | 17.1 | 18.4 | 18.0 |
| Household wealth quintile |  |  |  |  |  |  | *** | *** | *** |
| First | 15.5 | 13.8 | 14.5 | 14.2 | 13.5 | 13.8 | 12.9 | 12.2 | 12.4 |
| Second | 18.4 | 21.1 | 20.1 | 18.3 | 21.0 | 20.0 | 17.7 | 20.5 | 19.5 |
| Third | 21.3 | 22.9 | 22.3 | 21.6 | 23.1 | 22.5 | 21.4 | 23.0 | 22.4 |
| Fourth | 24.0 | 21.9 | 22.7 | 24.2 | 21.9 | 22.8 | 24.9 | 22.1 | 23.1 |
| Fifth | 20.8 | 20.2 | 20.4 | 21.7 | 20.5 | 20.9 | 23.1 | 22.3 | 22.6 |
| State of residence |  |  |  |  |  |  | *** | *** | *** |
| Bihar | 47.9 | 36.9 | 41.0 | 47.6 | 37.1 | 41.0 | 50.8 | 39.0 | 43.3 |
| Uttar Pradesh | 52.1 | 63.1 | 59.0 | 52.4 | 62.9 | 59.0 | 49.2 | 61.0 | 56.7 |
| Number of respondents@ | 9,419 | 5,969 | 15,388 | 8,796 | 5,766 | 14,562 | 6,591 | 4,746 | 11,337 |

**@** excludes the sample of girls who were married at wave 1; ****** indicates that the distribution of the sample of currently enrolled adolescents differed with that of the overall sample at p<=0.01; ******* indicates that the distribution of the sample of currently enrolled adolescents differed with that of the overall sample at p<=0.001
