# Supplementary material for: Learning deficits and early school leaving: Evidence from a longitudinal study in India
Source: PLoS One. 2025 Nov 18;20(11):e0336850. doi: 10.1371/journal.pone.0336850 (PMC12626265; doi:10.1371/journal.pone.0336850)
Supplement: S2 Table — (DOCX) [file pone.0336850.s002.docx]

**S2 Table: Number of respondents who were interviewed and who took learning assessments at wave 1 and wave 2, Bihar and Uttar Pradesh, 2015-16 and 2018-19**

|  | **Interviewed in 2015-16** | | | **Re-interviewed in 2018-19** | | |
| --- | --- | --- | --- | --- | --- | --- |
|  | **Girls** | **Boys** | **Total** | **Girls** | **Boys** | **Total** |
| Number of respondents@ | 9419 | 5969 | 15388 | 7607 [80.8%] | 4428 [74.1%] | 12035 [78.2%] |
| Number of respondents ever enrolled in school | 8796 | 5766 | 14562 | 7159 [81.4%] | 4317 [74.9%] | 11476 [78.8%] |
| Number of respondents who were currently enrolled in school, including distance learning at wave 1 | 6591 | 4746 | 11337 | 5493 [83.3%] | 3676 [77.5%] | 9169 [80.9%] |
| Took learning assessment | 8796 | 5766 | 14562 | 6826 [77.6%] | 4173 [72.4%] | 10999 [75.5%] |

**@** excludes the sample of girls who were married at wave 1; values in the parentheses indicate follow-up rates
