# Supplementary material for: Learning deficits and early school leaving: Evidence from a longitudinal study in India
Source: PLoS One. 2025 Nov 18;20(11):e0336850. doi: 10.1371/journal.pone.0336850 (PMC12626265; doi:10.1371/journal.pone.0336850)
Supplement: S3 Table — (DOCX) [file pone.0336850.s003.docx]

**S3 Table: Results from the probit regression analysis of attrition bias**

| **Background characteristics** | **Ever enrolled at wave 1** | | | **Currently enrolled at wave 1** | | |
| --- | --- | --- | --- | --- | --- | --- |
|  | **Girls (Coefficient)** | **Boys (Coefficient)** | **Total (Coefficient)** | **Girls (Coefficient)** | **Boys (Coefficient)** | **Total (Coefficient)** |
| Currently studying (Ref.No) | 0.312*** [0.20 – 0.42] | 0.553*** [0.47 – 0.63] | 0.451*** [0.39 – 0.51] | --- | --- | --- |
| Years of schooling completed | -0.004 [-0.02 – 0.01] | -0.050*** [-0.06 – -0.04] | -0.036*** [-0.04 – -0.03] | -0.008 [-0.02 – 0.01] | -0.067*** [-0.08 – -0.06] | -0.051*** [-0.06 – -0.04] |
| Muslim: Religion (Ref. Hindu) | -0.164*** [-0.27 – -0.06] | -0.187*** [-0.27 – -0.10] | -0.156***[-0.22 – -0.09] | -0.212*** [-0.33 – -0.09] | -0.212*** [-0.31 – -0.12] | -0.210*** [-0.28 – -0.14] |
| Caste (Ref. General castes) |  |  |  |  |  |  |
| Scheduled castes/tribes | 0.201** [0.06 – 0.34] | 0.135** [0.04 – 0.23] | 0.138*** [0.06 – 0.22] | 0.239*** [0.08 – 0.40] | 0.135** [0.03 – 0.24] | 0.149*** [0.06 – 0.24] |
| Other backward castes | 0.152** [0.04 – 0.26] | 0.150*** [0.07 – 0.23] | 0.142*** [0.08 – 0.21] | 0.159*** [0.04 – 0.28] | 0.163*** [0.08 – 0.25] | 0.157*** [0.09 – 0.23] |
| Wealth quintiles | 0.002 [-0.00 – 0.01] | 0.01*** [0.00 – 0.01] | 0.006*** [0.00 – 0.01] | 0.001 [-0.01 – 0.01] | 0.007** [0.00 – 0.01] | 0.005** [0.00 – 0.01] |
| Urban: Place of residence (Ref. Rural) | -0.318*** [-0.43 – -0.21] | -0.250*** [-0.34 – -0.16] | -0.248*** [-0.31 – -0.18] | -0.326*** [-0.45 – -0.21] | -0.281*** [-0.38 – -0,19] | -0.269*** [-0.34 – -0.20] |
| Bihar: State (Ref. Uttar Pradesh) | 0.199*** [0.10 – 0.29] | 0.044 [-0.02 – 0.11] | 0.123*** [0.07 – 0.17] | 0.196*** [0.09 – 0.30] | 0.031[-0.04 – 0.10] | 0.110*** [0.05 – 0.17] |
| Constant | 0.711*** [0.50 – 0.92] | 0.507*** [0.36 – 0.65] | 0.587*** [0.47 – 0.71] | 1.097*** [0.87 – 1.32] | 1.203*** [1.05 – 1.36] | 1.165*** [1.04 – 1.29] |
| Number of respondents | 8,796 | 5,766 | 14,562 | 6,591 | 4,746 | 11,337 |

Note: ***p<=0.001; **p<=0.01; *p<=0.05
