# Supplementary material for: Learning deficits and early school leaving: Evidence from a longitudinal study in India
Source: PLoS One. 2025 Nov 18;20(11):e0336850. doi: 10.1371/journal.pone.0336850 (PMC12626265; doi:10.1371/journal.pone.0336850)
Supplement: S4 Table — (DOCX) [file pone.0336850.s004.docx]

**S4 Table: Description for explanatory variables used in the multivariate analyses**

| **Variables** | **Definition and response categories** | **Discrete-time hazard model** | **Fixed effects model** |
| --- | --- | --- | --- |
| Age | In completed years | √ | √ |
| Sex | Female/male | √ |  |
| Engagement in paid work | Engaged in paid work in the year preceding the interview (Yes/No) | √ | √ |
| Marital status | Got married during the inter-survey period (Yes/No) | √ | √ |
| Decision-making say | Had some say in all decisions; items for those who were aged 10-14 years at wave 1 were years of schooling they should have and their choice of friends and items for those who were aged 15-19 years were years of schooling they should have, major household purchases, and whether to work or stay at home | √ | √ |
| Freedom of movement | Allowed to visit unescorted two out of three locations - a shop/market or a friend/relative within their village/ ward, a shop/ market or a friend/relative outside their village/ward, and a programme (mela, sports event, adolescent group meetings) within their village/ward | √ | √ |
| Gender-role attitudes | Scored 4 or 5 on the index of gender role attitudes; the statements referred to: the relative importance of educating boys versus girls and boys’ sharing household chores with their sisters (for those aged 10-14 years at wave 1); fathers and mothers sharing chores related to childcare and a girl having a male friend (for those aged 15-19 years); and girls’ interest in being teased by boys, girls’ right to be involved in decisions related to timing of their marriage, and fathers’/husbands’ perceived right to decide about spending household money (for both groups). | √ | √ |
| Age at first-time enrolment | Age when enrolled in school for the first time in completed years | √ |  |
| Household wealth | Household wealth index based on ownership of selected durable goods and amenities with possible scores ranging from 0 to 57 | √ | √ |
| Religion | Hindu/ Muslim and others^1^ | √ |  |
| Caste | Scheduled caste or tribe, other backward caste and general caste | √ |  |
| Mother’s education | Mothers who were literate (Yes/No) | √ |  |
| Parent-child communication | An additive index that captured the number of topics that adolescents discussed with their parent/s in the year preceding the interview - school performance, friendship and physical changes during adolescence or how pregnancy occurs) | √ | √ |
| Gender discriminatory experiences at home | Experienced gender discriminatory practices at home where parents favoured sons over daughters in any of the following (the quantity or quality of food items given, the amount of pocket money given and the type of school in which they were enrolled)^2^ (Yes/No) | √ | √ |
| Type of school attended | Government/ private school | √ | √ |
| Private coaching | Received private coaching in the month preceding the interview (Yes/No) | √ | √ |
| Enrolled in schools with basic amenities | Attended school with basic amenities such as drinking water, functional toilet, playground and library (Yes/No) | √ | √ |
| Place of residence | Rural/ urban | √ | √ |
| State | Bihar/ Uttar Pradesh | √ |  |

0.6% girls and 0.3% boys belonged to other religions. 2 Questions on gender discriminatory practices at home, i.e., whether their parents favoured them (among boys) or discriminated against them (among girls) vis-à-vis their opposite-sex siblings, were posed to those who reported co-residing with opposite-sex siblings who were up to three years younger or older than the respondent; those respondents who were not eligible for these questions were considered not to have experienced gender discriminatory practices.
